# The Association Between Fathers’ Self-assessment of Their Own Parenting and Mothers’ Recognition of Paternal Support: A Municipal-Based Cross-Sectional Study

**DOI:** 10.2188/jea.JE20200108

**Published:** 2021-12-05

**Authors:** Toshihiro Terui, Kazuki Yoshida, Mie Sasaki, Michio Murakami, Aya Goto

**Affiliations:** 1 International Community Health, Graduate School of Medicine, Fukushima Medical University, Fukushima, Japan; 2 Center for Integrated Science and Humanities, Fukushima Medical University, Fukushima, Japan; 3 Faculty of Humanities, Saitama Gakuen University, Saitama, Japan; 4 Department of Health Risk Communication, Fukushima Medical University School of Medicine, Fukushima, Japan

**Keywords:** father involvement, coparenting, family relations, parenting time

## Abstract

**Background:**

Discrepancies between parents’ reports of paternal parenting have been gaining attention, but epidemiological evidence is scarce in Asia. This study aimed to clarify agreement/discrepancy between paternal and maternal recognition of paternal parenting and the association between actual paternal parenting time and background factors.

**Methods:**

Data from couples whose children attended 4-month child health check-ups in Fukushima City were analyzed (*N* = 509). Based on paternal recognition of paternal parenting (PRPP) and maternal recognition of paternal support (MRPS), couples were classified into four groups. Each group’s paternal household work and parenting time were analyzed. Univariable and multivariable analysis were performed to investigate the association between agreement/discrepancy and background factors of children and parents.

**Results:**

Frequency of positive agreement (PRPP+ and MRPS+) was 83.9%, whereas negative agreement (PRPP− and MRPS−) was 2.6%. As for discrepancy, PRPP+ and MRPS− was 8.4% and PRPP− and MRPS+ was 5.1%. Fathers’ total median parenting time was 2 (weekdays) and 6 (weekends) hours, and showed significant differences among the four groups. Multivariable analysis revealed that compared to positive agreement, maternal mental health condition and pregnancy intention were significantly associated with the discrepancy PRPP+ and MRPS−, paternal mental health condition and marital satisfaction with the discrepancy PRPP− and MRPS+, and maternal mental health condition with negative agreement.

**Conclusions:**

We identified differences in parenting time and mental health characteristics among couples depending on agreement/discrepancy in recognition of paternal parenting. Assessing both parents’ profiles is necessary in clinical practice to promote paternal participation in childcare.

## INTRODUCTION

Japanese mothers tend to experience physical and mental fatigue in the early postpartum period because, in part, they receive little support from their husbands.^1^ Maternal mental distress after child birth leading to marital difficulties is gaining social attention, and has been referred to as the “postpartum crisis” by national media reports.^2^ To construct more effective parenting support policies and programs in clinical practice, maternal and child health professionals should rethink ways to promote and support coparenting. Currently, Japanese policies focus mainly on supporting women and their child rearing burden based on the male breadwinner model.^3^ More attention needs to be drawn to fathers’ responsibilities in parenting.

During the past two decades, the importance of paternal parenting has been emphasized worldwide due to the increasing significance of women’s role in the workforce and society and diversifying family structures.^4^ It has been reported that the presence of fathers is associated with children’s development, academic achievements, psychological adjustments, and anger management.^5^ The roles of fathers include not only direct contact with children, but also indirect involvement, such as economic and emotional support.^5^ Moreover, paternal parenting helps improve the quality of marital relationships.^5^ To date, multifarious variables have been identified as predictive of paternal parenting: children’s characteristics (sex), paternal characteristics (age, income, history of depression, depressive symptoms, greater human capital, self-efficacy), maternal characteristics (race, depressive symptoms), and family characteristics (marital and cohabiting status, marital relationship, cooperative coparenting).^6^^–^^13^

The best way to assess paternal parenting remains under debate. Coley and his colleagues^14^ showed that the paternal parenting reported by fathers themselves had more consistent predictive validity for children’s cognitive skills than paternal parenting as reported by their partners. Other studies by the same authors^15^ and Mikelson^16^ reported explicit discrepancies between mothers’ and fathers’ reports of paternal parenting. Furthermore, Charles et al^17^ revealed more detailed discrepancy in the recognition of paternal parenting, and argued the discrepancy between parents’ recognition varied depending on the type of involvement by fathers. From mothers’ perspective, one study focusing on adolescent mothers’ depression showed there was a significant association between mothers’ perception/satisfaction toward their partners’ involvement and their mental status,^18^ which suggests mothers’ recognition of paternal parenting is linked to their emotional well-being. Existing evidence suggests a potential gap in parental recognition of paternal parenting and the importance of subjective recognition, as well as parental characteristics associated with the discrepancies in recognition of paternal parenting.^15^^,^^16^ However, there are still few studies on this topic, particularly in Asia. Future studies should reflect past evidence by acquiring information on both parents’ assessments of paternal parenting since reports from only one parent could possibly provide a skewed view of actual paternal parenting at home. Moreover, such efforts to understand within-couple discrepancies in how they perceive paternal parenting would encourage maternal and child health practitioners to pay closer attention to fathers as co-equal to mothers in caregiving, and in turn, empower fathers to be more involved.

According to the results of a child health survey published by The Japanese Society of Child Health in 2010, the proportion of fathers who actively play with their children (from 49% in 2000 to 58% in 2010) and support their wives (from 65% in 2000 to 79% in 2010) is increasing.^19^ Nevertheless, paternal parenting and housework time in Japan—about 1 hour 23 minutes per day—still remains shorter than in other developed countries: 3 hours 10 minutes in the United States, 2 hours 46 minutes in the United Kingdom, and 3 hours in Germany.^20^ McHale et al^21^ indicated that caregivers need to spend 8–10 hours each day with their child to establish a core bond. Researchers in Japan have suggested efforts to improve marital communication as a motivating factor of paternal parenting.^22^ For example, a recent trial in Japan aiming to improve fathers’ empathy toward their partners reported positive effects on preventing maternal postpartum depression.^23^ Stepping beyond the traditional male breadwinner model,^3^ discussing the agreement and discrepancy between paternal and maternal recognition of paternal parenting could provide scientific evidence for developing a couple-based family support model in Japan. Moreover, this could provide a path for promoting paternal parenting in other countries, especially Asian countries where little work has been done to investigate both parents’ recognition of paternal parenting.

In this study, we aimed to clarify the agreement and discrepancy between paternal and maternal recognition of paternal parenting and support, and the association with actual paternal parenting time and background factors.

## METHODS

### Study design and participants

**Figure 1.**
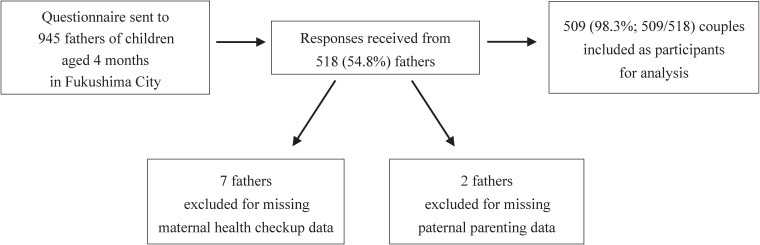
Recruitment and process of enrollment of study participants

This was a community-based cross-sectional study. The survey targeted couples of children who attended health checkups for 4-month-old children between October 2017 and March 2018 in Fukushima City. We sent questionnaires to 945 fathers, and responses were later collected at the time of 4-month health checkups from 518 (54.8%) fathers. We also copied maternal, child, and household data from the child health checkup files. In most cases, mothers completed the checkup forms. In our analysis, seven cases with missing data on mothers and two with missing data on fathers were excluded. Data for 509 (98.3%; 509/518) couples were included in the final analyses (Figure 1).

### Measures

We compared responses to questions asking fathers about their own involvement in parenting and household work and mothers about their recognition of support from their husbands. Following one evaluation indicator from the national maternal and child health plan (Healthy Parents and Children 21), “the proportion of fathers who actively take care of their children”, we asked fathers the question, “Are you engaged in parenting?”. There were four answer options: “frequently”, “sometimes”, “almost never”, and “no”. We dichotomized these answers with the former two responses defined as “yes” and the latter two as “no”. To mothers, we asked them to select people whom they seek for emotional and instrumental support from the following list: “partner”, “parents or siblings”, “friends”, “neighbors”, and “governmental and private consulting or services”. If mothers did not select “partner” for both, they were classified as not recognizing paternal support.

To assess the actual extent of paternal parenting and compare agreement and discrepancy between self-recognition of paternal parenting and maternal reliance on paternal support, we further asked fathers about their involvement in household work and parenting time with the question “How long do you spend on household work, parenting, and child rearing a day? Please answer both for weekdays and weekends”. This question was taken from the Longitudinal Survey of Adults in the 21st Century.

Other items for exploratory analysis of factors associated with recognitional discrepancy were as follows: children’s characteristics (sex, birth weight, order of birth), family structure (whether the household included the nuclear family or extended family), father’s characteristics (age, employment status, work demands, physical and mental health condition, pregnancy intention, marital satisfaction), and mother’s characteristics (age, employment status, physical and mental health condition, pregnancy intention). Most of these are well-known factors associated with paternal parenting or coparenting.^6^^–^^8^^,^^10^^–^^12^^,^^21^^,^^24^

We asked the following question about pregnancy intention to both parents, “How did you feel when you learned about the pregnancy with this child?”. The five answer options were “very happy”, “unintended but happy”, “unintended and confused”, “troubled”, and “no emotion”. This is a commonly asked question during child health checkups in order to screen child abuse.

We measured parental mental health condition with the questions from the Minami-Tama method of child abuse screening developed by the Tokyo Minamitama Health Center. Parents were asked about their mental health condition with the question, “How is your mental health condition?”. The three answer options were “good”, “not sure”, and “not good”. We dichotomized the answers by classifying those other than “good” as poor mental health condition. As for physical health, we used data from the health checkup sheet with answer options “good” and “poor”.

We measured work demands with occupational stress measures developed by Kawakami.^25^ We used three categories of work demands (having an extreme amount of work, being unable to complete work within the allotted time, and having to work as hard as possible). For each question, fathers answered using the following four options: “strongly agree”, “agree”, “disagree”, and “strongly disagree”. We calculated the means of the three quantified scores. Fathers with higher scores had lower workload demands.

### Analysis

Our analysis flow was to first identify the distribution of four agreement/disagreement categories, second to confirm its association with the level of paternal parenting (self-reported parenting time), and third to explore the background factors of these categories. First, we grasped the distribution of the combination of paternal self-recognition of their own parenting (paternal recognition of paternal parenting [PRPP]) and maternal recognition of their husbands’ (paternal) emotional/instrumental support (maternal recognition of paternal support [MRPS]). Then, participants were classified into four groups (Group A: PRPP+ and MRPS+, Group B: PRPP+ and MRPS−, Group C: PRPP− and MRPS+, and Group D: PRPP− and MRPS−). In other words, Group A was the positive agreement group, Group D was the negative agreement group, and Groups B and C were discrepancy groups. Second, we analyzed differences in time spent on household work and parenting (weekdays and weekends) between Group A and each of the other three groups using the Mann-Whitney U-test. Third, we conducted univariable analyses between Group A and each of the other three groups, in which either or both parents did not recognize paternal parenting. Finally, using a logistic regression model, we conducted multivariable analysis between Group A and each of other three groups, by entering significant items in the univariable analyses. We adopted the Bonferroni method to adjust *P*-values for the assessment of parenting time and univariable analysis. SPSS version 25 (IBM Corp, Armonk, NY, USA) was used for the analyses.

### Ethical considerations

This study was a joint enterprise in which researchers cooperated with the Fukushima City municipal government. The study was approved by the ethics committee of Fukushima Medical University (No. 3570). We sent letters requesting participation in this study to target families and returning a completed questionnaire was taken as providing informed consent to participate in the study.

## RESULTS

### Participant characteristics

Basic characteristics of the participants are shown in Table 1. Almost half of the children were male (*n* = 264, 52.0%) and 52.7% were first-borns (*n* = 268). The mean age of the fathers was 33.07 (standard deviation [SD], 6.15) years and most of them were employed (*n* = 502, 99.2%). All fathers had good physical health condition, and 24.4% had poor mental health condition (*n* = 124). The mean age of the mothers was 30.97 (SD, 4.79) years and 55.0% were employed (*n* = 279). Poor physical health condition was identified in 0.6% of the mothers (*n* = 3), and 15.3% had poor mental health condition (*n* = 77).

**Table 1.** Basic characteristics of participants (*N* = 509)

		*N*	%
Characteristics of child			
Sex	Boy	264	52.0
	Girl	244	48.0
Birth order	First	268	52.7
	On and after second child	241	47.3
Birth weight	≥2,500 g	467	91.7
	<2,500 g	42	8.3

Family structure	Nuclear family	420	84.3
	Extended family	78	15.7
Characteristics of father			
Age	Mean (SD)	33.07 (6.15)	
	<30 years	137	27.0
	≥30 years	370	73.0
Employed	Yes	502	99.2
	No	4	0.8
Work demand score	Mean (SD)	1.86 (0.64)	
Physical health condition	Good	499	100.0
	Poor	0	0.0
Mental health condition	Good	384	75.6
	Poor	124	24.4
Pregnancy intention^a^	“Happy”	480	96.2
	Except “Happy”	19	3.8
Marital satisfaction	Satisfied	496	98.0
	Not satisfied	10	2.0
Characteristics of mother			
Age	Mean (SD)	30.97 (4.79)	
	<30 years	194	38.1
	≥30 years	315	61.9
Employed	Yes	279	55.0
	No	228	45.0
Physical health condition	Good	491	99.4
	Poor	3	0.6
Mental health condition	Good	427	84.7
	Poor	77	15.3
Pregnancy intention	“Happy”	482	94.7
	Except “Happy”	27	5.3

SD, standard deviation.

^a^Pregnancy intention: The category “except happy” includes “unintended and confused”, “troubled”, and “no emotion”.

### Recognition of each parent

In 427 (83.9%) couples, fathers recognized their own parenting and their partners were aware of their emotional/instrumental support (Table 2). We classified these couples as Group A. On the other hand, neither partner recognized paternal parenting and support in 13 (2.6%) couples, which we classified as Group D.

**Table 2.** Distribution of parental recognition of paternal parenting and support

		Maternal recognition of paternal support (MRPS)^a^		Total

		Yes	No	
Paternal recognition of paternal parenting (PRPP)	Yes	Group A 427 (83.9%)	Group B 43 (8.4%)	509
	No	Group C 26 (5.1%)	Group D 13 (2.6%)	

Group A: PRPP+ and MRPS+, Group B: PRPP+ and MRPS−, Group C: PRPP− and MRPS+, Group D: PRPP− and MRPS−.

^a^MRPS: If mothers did not select “partner” as a person whom they seek for emotional or instrumental support, they were classified as not recognizing paternal support.

### Comparison of self-reported household work and parenting time

There were significant differences in median time for weekday household work and parenting between Group A and Group C (*P* < 0.001) and Group D (*P* = 0.003) (Table 3). There were also significant differences in median time for weekend household work and parenting between Group A and the other three groups (Group B, *P* = 0.027; Group C, *P* < 0.001; Group D, *P* = 0.003). No significant difference in median time for weekday household work and parenting was found between Groups A and B (*P* = 0.138).

**Table 3.** Paternal self-reported household work and parenting time of each groups

		Parental recognition of paternal parenting and support^a^						
	
	Total	A	B	B vs A	C	C vs A	D	D vs A
	(*N* = 509)	(*N* = 427)	(*N* = 43)		(*N* = 26)		(*N* = 13)	
	
	Median (Min, Max)	Median (Min, Max)	Median (Min, Max)	*P*-value^b^	Median (Min, Max)	*P*-value^b^	Median (Min, Max)	*P*-value^b^
	
Household work and parenting time on weekdays, hours	2 (0, 12)	2 (0, 12)	1 (0, 6)	0.138	1 (0, 4)	<0.001^***^	0.5 (0, 3)	0.003^**^
Household work and parenting time on weekends, hours	6 (0, 24)	6 (0.5, 24)	4 (0.5, 12)	0.027^*^	2 (0, 12)	<0.001^***^	2 (0, 9)	0.003^**^

MRPS, maternal recognition of paternal support; PRPP, parental recognition of paternal parenting and support.

^a^A: PRPP+ and MRPS+, B: PRPP+ and MRPS−, C: PRPP− and MRPS+, D: PRPP− and MRPS−.

^b^Mann-Whitney U test was adopted by using Bonferroni correction.

^*^: *P* < 0.05, ^**^: *P* < 0.01, ^***^: *P* < 0.001.

### Univariable and multivariable analysis of participant characteristics

#### Groups A and B

Discrepancy in recognition of paternal parenting and support between parents was significantly associated with mothers’ mental health condition and pregnancy intention (Table 4). In the multivariable analysis, both of these variables remained significantly associated with discrepancy in recognition of paternal parenting between parents (mothers’ mental health condition: odds ratio [OR] 2.64; 95% confidence interval [CI], 1.25–5.60; mothers’ pregnancy intention: OR 5.02; 95% CI, 1.88–13.44) (Table 5).

**Table 4.** Univariable analysis of characteristics of child and parents

		Parental recognition of paternal parenting and support^a^						
		*N* (%)						

Characteristics of child and parents		A	B	B vs A	C	C vs A	D	D vs A
		(*N* = 427)	(*N* = 43)	*P*-value^b^	(*N* = 26)	*P*-value^b^	(*N* = 13)	*P*-value^b^
Characteristics of children								
Sex	Boy	217 (50.9)	27 (62.8)	0.414	14 (53.8)	1	6 (46.2)	1
	Girl	209 (49.1)	16 (37.2)		12 (46.2)		7 (53.8)	
Birth order	First	225 (52.7)	26 (60.5)	0.990	13 (50.0)	1	4 (30.8)	0.357
	On and after second	202 (47.3)	17 (39.5)		13 (50.0)		9 (69.2)	
Birth weight	≥2,500 g	393 (92.0)	39 (90.7)	1	23 (88.5)	1	12 (92.3)	1
	<2,500 g	34 (8.0)	4 (9.3)		3 (11.5)		1 (7.7)	
Family structure	Nuclear family	353 (84.4)	35 (85.4)	1	20 (76.9)	0.843	12 (92.3)	1
	Extended family	65 (15.6)	6 (14.6)		6 (23.1)		1 (7.7)	
Characteristics of fathers								
Age	<30 years	110 (25.8)	17 (40.5)	0.126	7 (26.9)	1	3 (23.1)	1
	≥30 years	316 (74.2)	25 (59.5)		19 (73.1)		10 (76.9)	
Occupation	Yes	420 (99.1)	43 (100.0)	1	26 (100.0)	1	13 (100.0)	1
	No	4 (0.9)	0 (0.0)		0 (0.0)		0 (0.0)	
Work demands score	Mean (SD)	1.89 (0.65)	1.83 (0.57)	1	1.63 (0.61)	0.102	1.64 (0.55)	0.696
Mental condition	Good	333 (78.2)	31 (72.1)	1	14 (53.8)	0.012^*^	6 (46.2)	0.039^*^
	Poor	93 (21.8)	12 (27.9)		12 (46.2)		7 (53.8)	
Pregnancy intention	“Happy”	409 (97.4)	37 (90.2)	0.108	22 (88.0)	0.114	12 (92.3)	0.930
	Except “Happy”	11 (2.6)	4 (9.8)		3 (12.0)		1 (7.7)	
Marital satisfaction	Satisfied	423 (99.1)	41 (95.3)	0.291	22 (84.6)	0.003^**^	10 (100.0)	1
	Not satisfied	4 (0.9)	2 (4.7)		4 (15.4)		0 (0.0)	
Characteristics of mothers								
Age	<30 years	165 (38.6)	20 (46.5)	0.942	6 (23.1)	0.336	3 (23.1)	1
	≥30 years	262 (61.4)	23 (53.5)		20 (76.9)		10 (76.9)	
Occupation	Yes	239 (56.1)	18 (42.9)	0.300	14 (53.8)	1	8 (61.5)	1
	No	187 (43.9)	24 (57.1)		12 (46.2)		5 (38.5)	
Physical condition	Good	411 (99.3)	42 (100.0)	1	25 (100.0)	1	13 (100.0)	1
	Poor	3 (0.7)	0 (0.0)		0 (0.0)		0 (0.0)	
Mental condition	Good	371 (87.5)	29 (70.7)	0.009^**^	21 (80.8)	1	6 (46.2)	0.003^**^
	Poor	53 (12.5)	12 (29.3)		5 (19.2)		7 (53.8)	
Pregnancy intention	“Happy”	411 (96.3)	36 (83.7)	0.009^**^	23 (88.5)	0.264	12 (92.3)	1
	Except “Happy”	16 (3.7)	7 (16.3)		3 (11.5)		1 (7.7)	

MRPS, maternal recognition of paternal support; PRPP, parental recognition of paternal parenting and support.

^a^A: PRPP+ and MRPS+, B: PRPS+ and MRPS−, C: PRPP− and MRPS+, D: PRPP− and MRPS−.

^b^Mann-Whitney U test was adopted for analysis of work demands score, and Chi-square test and Fisher’s’ exact test for analysis other categorical variables by using Bonferroni correction.

^*^: *P* < 0.05, ^**^: *P* < 0.01.

**Table 5.** Multivariable analysis of characteristics of parents

Items		B vs A^a^		C vs A^a^		D vs A^a^	

		OR	95% CI	OR	95% CI	OR	95% CI
Characteristics of fathers							
Mental condition	Good			Ref		Ref	
	Poor			2.55	1.10–5.94	2.94	0.92–9.44
Marital satisfaction	Satisfied			Ref			
	Not satisfied			14.19	3.17–63.54		
Characteristics of mothers							
Mental condition	Good	Ref				Ref	
	Poor	2.64	1.25–5.60			6.38	1.99–20.45
Pregnancy intention	“Happy”	Ref					
	Except “Happy”	5.02	1.88–13.44				

CI, confidence interval; MRPS, maternal recognition of paternal support; OR, odds ratio; PRPP, parental recognition of paternal parenting and support.

A: PRPP+ and MRPS+, B: PRPP+ and MRPS−, C: PRPP− and MRPS+, D: PRPP− and MRPS−.

^a^Logistic regression analysis was adopted for the analysis to calculate risk of having a discrepancy (B, C, or D). Significant items in the univariable analyses were entered into the model.

^*^: *P* < 0.05, ^**^: *P* < 0.01.

#### Groups A and C

Discrepancy between self-recognition of paternal parenting and maternal reliance on paternal support was significantly associated with fathers’ mental health condition and fathers’ marital satisfaction (Table 4). In the multivariable analysis, both of these variables remained significantly associated with discrepancy in recognition of paternal parenting between parents (fathers’ mental health condition: OR 2.55; 95% CI, 1.10–5.94; fathers’ marital satisfaction: OR 14.19; 95% CI, 3.17–63.54) (Table 5).

#### Groups A and D

Parental recognitional discrepancy was significantly associated with fathers’ mental health condition and mothers’ mental health condition (Table 4). In the multivariable analysis, only mothers’ mental health condition remained significantly associated with parents’ negative agreement of paternal parenting and support (OR 6.38; 95% CI, 1.99–20.45) (Table 5).

## DISCUSSION

This is the first study in Japan to report on the gap between self-recognition of paternal parenting and maternal reliance on paternal support. We confirmed that there were significant differences in self-reported time spent on household work and parenting between Group A (positive agreement group) and each of the other three groups (discrepancy groups and negative agreement group), except for on weekdays between Groups A and B. We further identified that parents’ mental health condition, pregnancy intention, and marital satisfaction were associated with parents’ discrepancy and negative agreement on paternal parenting.

### Recognition of paternal parenting and parenting time

There were significant differences in the average self-reported parenting time between Group A and each of the other three groups. Among the few existing reports focusing on parenting time, McHale et al^21^ suggested that parents or caregivers need to assure 8–10 hours are spent each day on parenting to establish a strong bond with their children. In terms of the effects of parenting time on children, Cabaj et al^26^ reported that adequate parenting time could be a protective factor against high risk of behavioral problems in children. These previous studies showed the significance of grasping parenting time for children’s present and future outcomes. In the present study, we reported that the length of self-reported paternal parenting time differed based on parental recognitional discrepancies of paternal parenting. Our study suggests that grasping both PRPP and MRPS may predict fathers’ tendencies regarding parenting time.

The average parenting time of the fathers in the present study was insufficient if we adopt McHale’s suggestion.^21^ However, close to 90% of mothers relied on their partners for emotional/instrumental support, indicating a low expectation in Japan toward fathers in terms of time allocated to parenting on a daily basis. This further suggests that we should consider the feasibility of extending paternal parenting time in current Japanese society. According to O’Hara et al,^27^ the association between more parenting time and the father-child relationship is not as simple as one might think, for a quadratic relationship between parenting time and paternal parenting quality exists. Along the same line, another study showed that greater paternal parenting time and lower paternal parenting quality were associated with negative outcomes in children.^28^ Further similar research is warranted in Japan to investigate subsequent effects of both paternal parenting time and “quality” to explore the suitable balance of shared responsibilities of both genders both inside and outside the home.

### Factors associated with parental discrepancy and negative agreement in paternal parenting

#### Group A with positive agreement and Group B with discrepancy (PRPP+ and MRPS−)

Maternal pregnancy intention of not being “happy” and poor mental health condition remained significantly associated with parental recognitional discrepancy of paternal parenting in cases in which the father positively reported their parenting, but the mother did not agree. Previous reports revealed an association between unintended pregnancy and maternal depression and various parenting behaviors.^29^^–^^31^ Our study indicated that pregnancy intention was associated with not only mothers’ own parenting behavior, but also their recognition of paternal support.

Contrary to mothers’ low recognition of paternal support in Group B, father-reported parenting time did not differ between groups. This may be attributable to two possible hypotheses related to mothers’ mental health condition. First, we might be able to apply a compensatory/buffering model to this circumstance; namely, that fathers might become more involved with their child to compensate for their partners’ poorer mental health.^6^ Second, fathers’ overestimating their own parenting time might make their partners fall into poorer mental health. More specifically, social desirability bias might exist in Japan. Regardless of the background pathway, we should pay attention to parental communication when providing parenting support to couples with unintended pregnancy experience. In support of this strategy, one study showed paternal parenting as a mediating factor between maternal depression and unintended pregnancy.^31^

#### Group A with positive agreement and Group C with discrepancy (PRPP− and MRPS+)

Fathers’ mental state (mental health condition and marital satisfaction) was associated with this type of discrepancy, in which their own recognition of parenting contribution was low. Previous research has shown that fathers tend to estimate their parenting higher than their partners.^15^^,^^16^ Likewise, among our study sample, the frequency of this type was lower than cases in which fathers over-estimated their parenting contribution. One explanation for the underlying reasons for fathers’ own low self-assessment is that marital relationship quality is known to be associated with Japanese fathers’ paternal postpartum depression,^32^ and further with paternal parenting, as reported from studies in other countries.^10^^,^^11^ William^33^ argued that the influence of fathers’ mental health status on coparenting was larger than mothers’ mental health. Despite its low frequency, health professionals need to support fathers when they face depression and a decline in childrearing confidence.

#### Group A with positive agreement and Group D with negative agreement (PRPP− and MRPS−)

For cases in which neither parent recognized or relied on paternal parenting or support, the mental health condition of both parents was the significantly associated factor in the univariable analysis, and only mothers’ mental health condition remained significant in the multivariable analysis. Recently, there is increased attention on the simultaneous occurrence of postpartum depression within couples,^34^ and the relationship between paternal postpartum depression and maternal depression has also been mentioned in Japan.^32^ Contrary to the compensatory/buffering models we previously introduced, spillover models may better fit Group D in the present study.^6^ Mothers’ poorer mental health might induce fathers to feel unpleasant, causing them to withdraw from parenting. When one partner is identified as having depression, we should also consider how it affects the other, for paternal depression induced by maternal depression has the possibility of evoking even poorer paternal parenting.

### Implications for clinical practice

Our present study indicated that poor mental health self-assessment in both parents and fathers’ low marital satisfaction affected recognition of paternal parenting. These results support previous studies’ suggestion that grasping the mental health condition of both parents is important.^32^^–^^36^ We suggest that health providers (eg, public health nurses) continue to strive to identify both parents’ health profiles at the time of perinatal care. Especially in Japan, it is usually only pregnant women or mothers who attend antenatal care or child health checkups. We suggest making paternal assessment part of routine care and promoting paternal participation in child care.

### Methodological limitations and future study

This study had some methodological limitations. First, we could not reveal the causal relationship between the discrepancy in parents’ recognition of paternal parenting and the associated variables because this research adopted a cross-sectional study design. Specifically, with regard to the spillover and compensatory/buffering models, time sequence design is necessary to clarify the relationship of mothers’ mental health with recognition and quantity of paternal parenting. Second, when it comes to fathers’ own reports of their time spent parenting, social desirability bias is inevitable. Recent fathering promotion policies and public support programs in Japan (eg, Iku-Men Project) have created the image of the “ideal father”, potentially leading fathers in the present study to provide responses that are socially favorable. In our survey, parents might have had a chance to see each other’s responses at home, which could have amplified the bias. Moreover, we did not ask fathers to differentiate time spent on parenting and household work. Applying a more detailed objective assessment of the time and types of paternal parenting is recommended for future investigations. Third, in the pathway of data collection, selection bias might have occurred. In Japan, it is mostly mothers who bring their babies to child health checkups. Therefore, fathers who responded to a non-routine survey were likely to be more actively engaged in parenting, and Group A might be over represented in the present study compared to the general public. Fourth, mothers’ information was obtained from routine health checkup sheets. Thus, we applied questionnaire items addressed to mothers asking about instrumental/emotional support provided by fathers as a pragmatic indicator reflecting maternal recognition of paternal parenting. In addition, our analysis lacked data on mothers’ satisfaction with the marital relationship, which is known to be associated with paternal parenting. Finally, because of the relatively small sample size in Group D, it is difficult for the authors to develop a conclusive discussion comparing positive agreement and negative agreement.

Despite these limitations, our current study showed differences between self-recognition of paternal parenting and maternal reliance on paternal support and their association with self-reported paternal parenting time and background characteristics among Japanese couples. Further investigations will be required to clarify how these discrepancies and paternal parenting time affect the quality of paternal parenting.
